# Labouring on decelerations: the fetal peripheral chemoreflex wins

**DOI:** 10.1113/JP272339

**Published:** 2016-09-01

**Authors:** D. A. Giussani

**Affiliations:** ^1^ Department of PhysiologyDevelopment & NeuroscienceUniversity of CambridgeDowning StreetCambridge CB2 3EGUK

Recordings of fetal heart rate provide the clinician with the only non‐invasive tool to continuously monitor fetal wellbeing during labour. By far, the most debated component of fetal heart rate monitoring during labour is the significance of fetal heart rate decelerations, which almost invariably occur in association with uterine contractions. Over the years, proposed possible triggers for these reductions in fetal heart rate have included fetal head compression, baroreflexes, chemoreflexes, Bezold–Jarisch reflexes and/or myocardial depression. In this issue of *The Journal of Physiology*, Lear *et al*. (2016) propose that a unified understanding of the physiology underlying intrapartum fetal heart decelerations is critical to improve their interpretation. The review reminds us that normal labour is associated with intermittent interruptions of uteroplacental gas exchange, such that ‘technically’ all babies experience some sort of asphyxia to a certain extent during labour. Although intrapartum fetal asphyxia is a term that is often avoided clinically, as it conjures thoughts of injury, it is a biochemical reality and it is useful for clinicians to understand the parameters underlying it. Reviewing and evaluating the available literature, Lear and colleagues suggest that, on balance, typical intrapartum decelerations are most likely the result of transient episodes of fetal hypoxia or fetal asphyxia and the consequent activation of the fetal peripheral chemoreflex response.

Historical experiments on the regulation of heart rate responses under hypoxic conditions in the adult individual, reported more than half a century ago, may shed additional light on the control of fetal heart rate during labour. In the early 1960s, Daly and Scott performed classic experiments on anaesthetised dogs that revealed an important interaction between ventilatory and cardiovascular responses mediated by the peripheral chemoreceptors. In very simple terms, they induced a chemoreflex by isolating both carotid bifurcation regions and perfusing them with hypoxic blood in dogs that were either mechanically ventilated or allowed to breathe spontaneously. Importantly, the carotid sinus pressure, measured with a mercury manometer, was maintained constant (De Burgh Daly & Scott, 1962). They reported that provided the rate and depth of breathing were maintained, stimulation of the carotid body chemoreceptors by hypoxic blood caused bradycardia and peripheral vasoconstriction. However, in contrast to the cardiovascular responses observed in the dogs with controlled ventilation, stimulation of the carotid bodies in spontaneously breathing animals that were allowed to hyperventilate led to the opposite responses: tachycardia and peripheral vasodilatation. Daly and Scott concluded that hypoxia elicits a primary cardiovascular carotid chemoreflex composed of a fall in heart rate and an increase in peripheral vascular resistance which becomes modified by hyperventilation and switches to a secondary cardiovascular carotid chemoreflex, yielding an increase in heart rate and a decrease in peripheral vascular resistance. During hyperventilation protective stretch receptors in the lungs increase their afferent discharge to the brainstem. This influences the cardiac and vasomotor centres, which respond by inhibiting both vagal discharge to the heart and sympathetic outflow to the peripheral circulations (De Burgh Daly *et al*. 1967). Thus, when oxygen availability can be increased by hyperventilation, vasodilatation and increased heart rate occur to promote systemic perfusion. Conversely, if oxygen availability is finite, the primary chemoreflex response of bradycardia and peripheral vasoconstriction persists to decrease oxygen consumption and/or make best use of the available oxygen supply.

The fetal cardiovascular responses to acute moderate fetal hypoxia or deep but brief episodes of fetal asphyxia include a vagally mediated fall in fetal heart rate and a fetal brain‐sparing circulatory reflex, diverting blood flow away from less essential vascular beds secondary to peripheral vasoconstriction (Giussani *et al*. 1993; Bennet *et al*. 1999). The falls in fetal heart rate during intrapartum fetal hypoxia or fetal asphyxia therefore are likely to represent examples of this hard‐wired cardiovascular strategic response to oxygen deprivation depending on the ability to expand our lungs or not. This teleological perspective strengthens Lear and colleagues’ assertion that the principal mechanism mediating the rapid falls in fetal heart rate during labour is an increase in peripheral chemoreflex activation in response to transient fetal hypoxia or fetal asphyxia, rather than increased vagal outflow in response to either head compression and/or mechanoreflexes, such as the arterial baroreflex or the Bezold–Jarisch reflex. The review also raises the point that there are many fundamental questions about fetal physiology remaining. For instance, within peripheral chemoreflex activation, the carotid rather than the aortic chemoreceptors seem to play a more prominent role in response to acute moderate fetal hypoxia (Giussani *et al*. 1993; Giussani, 2016). What about during severe and/or repeated fetal asphyxia? Is there greater recruitment of aortic chemoreceptor afferent fibre discharge during acute fetal asphyxia? Are there other receptors that have yet to be identified?

Unlike acute episodes of moderate fetal hypoxia, severe fetal asphyxia can cause fetal death or injury, particularly if sufficiently prolonged. Despite this risk, an important point to underline is that healthy fetuses being carried by mothers with normal uteroplacental perfusion can successfully adapt to the typical repeated but brief periods of asphyxia without injury, thanks to these robust, highly reliably peripheral chemoreflex responses (for reviews, see Gunn *et al*. 2001; Giussani, 2016). Lear and colleagues’ cutting edge review (Lear *et al*. 2016) elegantly illustrates to the reader that these fetal compensatory responses are physiological and not pathophysiological, and that it is the clinician's challenge to be able to identify when this changes and the fetus becomes decompensated. Lear and colleagues also encourage us to embrace the real nature of the fetal physiological responses to labour. This will help doctors and patients understand the limited predictive value of the fine detail analysis of fetal heart rate patterns that is still used in variable ways in different countries. More importantly, such understanding will help support the development of a more effective evidence base for successful clinical practice.

## Additional information

### Competing interests

None declared.

### Funding

The author is supported by the British Heart Foundation.
